# Circulating Tumor Cells: Back to the Future

**DOI:** 10.3389/fonc.2016.00275

**Published:** 2017-01-11

**Authors:** Giulia Gallerani, Pietro Fici, Francesco Fabbri

**Affiliations:** ^1^Biosciences Laboratory, Istituto Scientifico Romagnolo per lo Studio e la Cura dei Tumori (IRST) IRCCS, Meldola, Italy

**Keywords:** circulating tumor cells, CTC heterogeneity, EMT, cancer stem cell, CTC analysis

Circulating tumor cells (CTCs) are rare and heterogeneous cells found in the peripheral blood of cancer patients. They are supposed to be a central component of metastatic dissemination and have been correlated with prognosis, progression-free survival, and treatment efficacy in different solid tumors (1, 2). Despite CTC significance, studies conducted on them still present considerable issues. In particular, regarding to how CTCs should be investigated and to their actual metastatic competence and tumor heterogeneity reflecting dissimilar cancer cell subpopulations. Importantly, CTCs’ clinical utility has not been demonstrated and they cannot be utilized to guide therapeutic decisions. As reviewed by others (3), recent and currently ongoing clinical trials are trying to determine the predictive role of CTCs, but they are apparently failing to support definitive conclusions. Nevertheless, the results of these trials could shed a light on the real possibilities of CTCs. In our opinion, CTC troubleshootings will depend on what scientific community will explore about CTCs and the metastatic process. Only a deeper insight in base knowledge about CTCs and their role in tumor biology may help us in overcoming such hurdles.

A huge amount of efforts have been made to solve these problems. In the last decade, a number of technical solutions have been evaluated to study CTCs. Based on biological and/or physical properties (4), each approach owns strengths and weaknesses. Up to now, the only device that has reached a major confirmation by clinical evidence is the CELLSEARCH® System (5). Despite the pivotal results obtained with this system (6–9), its detection approach, based on epithelial markers only, fails to reflect all the potential CTC subpopulations, e.g., EpCAM-negative cells (10). Antigen-independent approaches that allow the identification of EpCAM-negative cells also (11–13) could eliminate the risk of underestimation of the dissimilar CTC populations; however, it could increase the risk of unspecific selection (14). Although the lack in technical standardization still hinders CTCs’ full translation in the clinical practice, these alternative methods could shed a light on the true nature of CTCs and pave the way to a clearer window into cancer biology and metastasis. In our opinion, in order to get further key insights into tumor aggressiveness, metastatic competence, heterogeneity, and resistance to treatment, we have to look back more deeply at base research, i.e., CTC-related epithelial–mesenchymal transition and stemness, CTC subpopulation/heterogeneity, and to CTC preclinical *ex vivo* studies.

It seems reasonable that CTC subsets and progression of metastasis could be enabled by EMT or by an EMT-like process (15). However, the involvement of EMT in metastatic dissemination is still debated (16, 17). It has been observed that EMT is not always needed for tumor cell motility (13) and recently, EMT was reported to be dispensable for metastasis, while contributing to chemoresistance (18, 19). Notably, not all the steps of EMT are necessary to establish an invasive phenotype of cancer cells (14). It has been postulated that EMT could be connected to cancer stemness (20) and that CTC population may comprise a subset of cells, with self-renewal, multi-potency, and tumor-initiating capabilities. Taking together, these aspects suggest two current needs for the CTC-research field. First, a detection approach more comprehensive than those established so far, able to catch all the different CTC subpopulations. Second, the necessity of a more in-depth analysis into the EMT regulatory networks during cancer initiation and progression (21). This approach will likely reveal the actual role of CTC subsets as “key players” involved in metastasis onset and progression (i.e., metastases-initiating CTCs), paving the way to innovative treatment regimens. Important technological improvements achieved in the field of genetic, genomic and transcriptomic analysis, as whole genome amplification (WGA), whole transcriptome amplification (WTA) for single cell, digital PCR (dPCR), and next-generation sequencing (NGS), could help to improve CTC research field and have to be accurately taken into consideration. Serial CTC molecular tumor profiling can facilitate the detection of primary (22) or acquired mechanisms of resistance to therapy, such as the emergence of ESR1 mutations in breast cancer or AR splicing variants in prostate cancer in response to targeted therapy (23, 24).

These last data indicate that monitoring regulatory networks and heterogeneity of CTCs, although still quite hampered by methodological issues, will offer significant clinical information about cancer progression, potential new therapeutic targets, and tumor sensitivity or resistance to therapy (25–29).

Circulating tumor cell population likely contains metastasis precursors, and their *ex vivo* culture represents a philosopher’s stone of translational-oncology research. *Ex vivo* culturing of CTCs may provide a powerful model of the metastatic cascade in basic research and a pivotal test for drug susceptibility/resistance in translational research (30).

In the last years, the CTC world’s leading groups had signed pioneering studies about *ex vivo* culturing of CTCs. *Ex vivo* CTC studies can be grouped as canonical *in vitro* culturing (11, 13, 30) and mice-incubator investigations (31, 32). Long-term CTC *in vitro* culturing were performed for the first time by the Marchetti’s and Maheswaran’s labs (11, 30), from blood of metastatic breast cancer patients. Zhang et al. detected and established CTC subpopulations targeted for brain metastases (11). These CTC cell lines were EpCAM negative, positive for a robust stem cell marker as ALDH1 and for Notch1, HER2, EGFR, and HPSE. Notably, CELLSEARCH® corresponding analyses revealed few or any “conventional” CTCs. Yu et al. generated CTC cell lines from consecutive single-patient blood draws (30). All the CTC cell lines were able to generate metastases in xenograft model proving their tumorigenic capability. Interestingly, both studies utilized a stem cell culture dedicated medium, strengthening the idea of the stem-like nature of CTCs. A CTC cell line was established from a metastatic colon cancer patient also (33). These tumorigenic cells owned the chromosomal aberrations observed in the primary tumor and were positive for ALDH and CD133 (stemness features).

A different *ex vivo* approach implied to expand CTCs into a xenograft model as a living “incubator.” This strategy consisted of a direct inoculation of enriched CTCs into immunodeficient mice without any *in vitro* passage. In an early study of Pretlow et al. (31), authors inoculated nucleated cells from 9 to 21 ml of blood from 14 treatment-refractory metastatic cancer patients (colon and prostate) into immunodepressed mice. Despite the lack of a CTC enrichment step, 3 out of 14 mice developed lung metastases. A more refined study was recently reported by Baccelli et al. (32). In this study, erythrocytes deprived blood from 3 out of 110 metastatic cancer patients formed different metastases (lung, liver, and bone) in recipient mice. Hodgkinson et al. (34) inoculated enriched CTCs in immunocompromised mice and established xenopatient models from six small-cell lung cancer patients. Xenopatient models mirrored patient responses to chemotherapy.

All these pioneering studies in *ex vivo* culturing of CTCs demonstrated how CTC basic research is important and necessary, from discovering specific genetic signature of resistance to mirroring patient response to therapy. More studies, however, have to be undertaken to expand our knowledge on CTC and translate their promises from bench to bedside.

A further critical matter that should be clarified is how many CTCs detected in few milliliters of blood may be representative of all relevant genetic and phenotypic heterogeneity of cancer cell with metastatic aptitude. A robust increase in the volume of blood analyzed will advantageously raise the number of detected CTCs, enabling the study of a higher more representative and informative number of tumor cells (35, 36).

In conclusion, we think that future base research studies on CTCs will be pivotal to move forward this field of investigation at both the preclinical and clinical levels. In order to progress toward the clinical utility of CTCs, biological data and technological improvements should be kept in mind and thoughtfully considered. To unravel CTC biology and to demonstrate their clinical value, a fully comprehensive CTC analysis approaches have to be achieved.

These advanced analyses combine: larger screened blood volume followed by CTC functional studies and deep next-generation “-omic” analysis. These steps could unveil CTC genetic heterogeneity, markers of resistance to therapy and identify actual metastases initiating CTCs. A new explanation of CTC at cellular and molecular level in both early and metastatic cancer stages (37) is now mandatory.

## Author Contributions

GG, PF, and FF were jointly responsible for writing the paper and agreed to its submission.

## Conflict of Interest Statement

The authors declare that the research was conducted in the absence of any commercial or financial relationships that could be construed as a potential conflict of interest.

## References

[1] Hanahan D Weinberg RA Hallmarks of cancer: the next generation. Cell (2011) 144:646–74. 10.1016/j.cell.2011.02.013 21376230

[2] Maheswaran S Haber DA Circulating tumor cells: a window into cancer biology and metastasis. Curr Opin Genet Dev (2010) 20:96–9. 10.1016/j.gde.2009.12.002 20071161 PMC2846729

[3] Alix-Panabières C Pantel K. Clinical applications of circulating tumor cells and circulating tumor DNA as liquid biopsy. Cancer Discov (2016) 6:479–91. 10.1158/2159-8290.CD-15-1483 26969689

[4] Alix-Panabières C Pantel K. Challenges in circulating tumour cell research. Nat Rev Cancer (2014) 14:623. 10.1038/nrc3686 25154812

[5] Joosse SA Gorges TM Pantel K Biology, detection, and clinical implications of circulating tumor cells. EMBO Mol Med (2014) 7(1):1–11. 10.15252/emmm.201303698 25398926 PMC4309663

[6] Cristofanilli M Budd GT Ellis MJ Stopeck A Matera J Miller MC Circulating tumor cells, disease progression, and survival in metastatic breast cancer. N Engl J Med (2004) 351:781–91. 10.1056/NEJMoa040766 15317891

[7] Bidard F-C Peeters DJ Fehm T Nolé F Gisbert-Criado R Mavroudis D Clinical validity of circulating tumour cells in patients with metastatic breast cancer: a pooled analysis of individual patient data. Lancet Oncol (2014) 15:406–14. 10.1016/S1470-2045(14)70069-5 24636208

[8] De Bono JS Scher HI Montgomery RB Parker C Miller MC Tissing H Circulating tumor cells predict survival benefit from treatment in metastatic castration-resistant prostate cancer. Clin Cancer Res (2008) 14:6302–9. 10.1158/1078-0432.CCR-08-0872 18829513

[9] Cohen SJ Punt CJ Iannotti N Saidman BH Sabbath KD Gabrail NY Relationship of circulating tumor cells to tumor response, progression-free survival, and overall survival in patients with metastatic colorectal cancer. J Clin Oncol (2008) 26:3213–21. 10.1200/JCO.2007.15.8923 18591556

[10] Grover PK Cummins AG Price TJ Roberts-Thomson IC Hardingham JE. Circulating tumour cells: the evolving concept and the inadequacy of their enrichment by EpCAM-based methodology for basic and clinical cancer research. Ann Oncol (2014) 25:1506–16. 10.1093/annonc/mdu018 24651410

[11] Zhang L Ridgway LD Wetzel MA Ngo J Yin W Kumar D The identification and characterization of breast cancer CTCs competent for brain metastasis. Sci Transl Med (2013) 5(180):180ra48 10.1126/scitranslmed.3005109 PMC3863909 23576814

[12] Vishnoi M Peddibhotla S Yin W Scamardo TA George GC Hong DS The isolation and characterization of CTC subsets related to breast cancer dormancy. Sci Rep (2015) 5:17533. 10.1038/srep17533 26631983 PMC4668355

[13] Aceto N Bardia A Miyamoto DT Donaldson MC Wittner BS Spencer JA Circulating tumor cell clusters are oligoclonal precursors of breast cancer metastasis. Cell (2014) 158:1110–22. 10.1016/j.cell.2014.07.013 25171411 PMC4149753

[14] Gkountela S Aceto N. Stem-like features of cancer cells on their way to metastasis. Biol Direct (2016) 11:33. 10.1186/s13062-016-0135-4 27457474 PMC4960876

[15] Rhim AD Mirek ET Aiello NM Maitra A Jennifer M Mccallister F EMT and dissemination precede pancreatic tumor formation. Cell (2013) 148:349–61. 10.1016/j.cell.2011.11.025 PMC3266542 22265420

[16] Yu M Bardia A Wittner BS Stott SL Smas ME Ting DT Circulating breast tumor cells exhibit dynamic changes in epithelial and mesenchymal composition. Science (2013) 339:580–4. 10.1126/science.1228522 23372014 PMC3760262

[17] Tarin D The fallacy of epithelial mesenchymal transition in neoplasia the fallacy of epithelial mesenchymal transition in neoplasia. Cancer Res (2005) 65:5996–6001. 10.1158/0008-5472.CAN-05-0699 16024596

[18] Zheng X Carstens JL Kim J Scheible M Kaye J Sugimoto H Epithelial-to-mesenchymal transition is dispensable for metastasis but induces chemoresistance in pancreatic cancer. Nature (2015) 527:525–30. 10.1038/nature16064 26560028 PMC4849281

[19] Fischer KR Durrans A Lee S Sheng J Li F Wong STC Epithelial-to-mesenchymal transition is not required for lung metastasis but contributes to chemoresistance. Nature (2015) 527:472–6. 10.1038/nature15748 26560033 PMC4662610

[20] Liu S Cong Y Wang D Sun Y Deng L Liu Y Breast cancer stem cells transition between epithelial and mesenchymal states reflective of their normal counterparts. Stem Cell Reports (2013) 2:78–91. 10.1016/j.stemcr.2013.11.009 24511467 PMC3916760

[21] De Craene B Berx G. Regulatory networks defining EMT during cancer initiation and progression. Nat Rev Cancer (2013) 13:97–110. 10.1038/nrc3447 23344542

[22] Marchetti A Del Grammastro M Felicioni L Malatesta S Filice G Centi I Assessment of EGFR mutations in circulating tumor cell preparations from NSCLC patients by next generation sequencing: toward a real-time liquid biopsy for treatment. PLoS One (2014) 9:e103883. 10.1371/journal.pone.0103883 25137181 PMC4138040

[23] Paoletti C Larios JM Muñiz MC Aung K Cannell EM Darga EP Heterogeneous estrogen receptor expression in circulating tumor cells suggests diverse mechanisms of fulvestrant resistance. Mol Oncol (2016) 10(7):1078–85. 10.1016/j.molonc.2016.04.006 27178224 PMC5423180

[24] Antonarakis ES Lu C Wang H Luber B Nakazawa M Roeser JC AR-V7 and resistance to enzalutamide and abiraterone in prostate cancer. N Engl J Med (2014) 371:1028–38. 10.1056/NEJMoa1315815 25184630 PMC4201502

[25] Stoecklein NH Hosch SB Bezler M Stern F Hartmann CH Vay C Direct genetic analysis of single disseminated cancer cells for prediction of outcome and therapy selection in esophageal cancer. Cancer Cell (2008) 13:441–53. 10.1016/j.ccr.2008.04.005 18455127

[26] Gallerani G Fabbri F Circulating tumor cells in the adenocarcinoma of the esophagus. Int J Mol Sci (2016) 17:1266 10.3390/ijms17081266 PMC5000664 27527155

[27] Heitzer E Auer M Gasch C Pichler M Ulz P Hoffmann EM Complex tumor genomes inferred from single circulating tumor cells by array-CGH and next-generation sequencing. Cancer Res (2013) 73:2965–75. 10.1158/0008-5472.CAN-12-4140 23471846

[28] Fabbri F Carloni S Zoli W Ulivi P Gallerani G Fici P Detection and recovery of circulating colon cancer cells using a dielectrophoresis-based device: KRAS mutation status in pure CTCs. Cancer Lett (2013) 335:225–31. 10.1016/j.canlet.2013.02.015 23419522

[29] Peeters DJE De Laere B Van den Eynden GG Van Laere SJ Rothé F Ignatiadis M Semiautomated isolation and molecular characterisation of single or highly purified tumour cells from CellSearch enriched blood samples using dielectrophoretic cell sorting. Br J Cancer (2013) 108:1358–67. 10.1038/bjc.2013.92 23470469 PMC3619252

[30] Yu M Bardia A Aceto N Bersani F Madden MW Donaldson MC Ex vivo culture of circulating breast tumor cells for individualized testing of drug susceptibility. Science (2014) 345:216–20. 10.1126/science.1253533 25013076 PMC4358808

[31] Pretlow TG Schwartz S Giaconia JM Wright AL Grimm HA Edgehouse NL Prostate cancer and other xenografts from cells in peripheral blood of patients. Cancer Res (2000) 60:4033–6. 10945604

[32] Baccelli I Schneeweiss A Riethdorf S Stenzinger A Schillert A Vogel V Identification of a population of blood circulating tumor cells from breast cancer patients that initiates metastasis in a xenograft assay. Nat Biotechnol (2013) 31:539–44. 10.1038/nbt.2576 23609047

[33] Cayrefourcq L Mazard T Joosse S Solassol JJ Ramos J Assenat E Establishment and characterization of a cell line from human circulating colon cancer cells. Cancer Res (2015) 75:892–901. 10.1158/0008-5472.CAN-14-2613 25592149

[34] Hodgkinson CL Morrow CJ Li Y Metcalf RL Rothwell DG Trapani F Tumorigenicity and genetic profiling of circulating tumor cells in small-cell lung cancer. Nat Med (2014) 20(8):897–903. 10.1038/nm.3600 24880617

[35] Fischer JC Niederacher D Topp SA Honisch E Schumacher S Schmitz N Diagnostic leukapheresis enables reliable detection of circulating tumor cells of nonmetastatic cancer patients. Proc Natl Acad Sci U S A (2013) 110:16580–5. 10.1073/pnas.1313594110 24065821 PMC3799344

[36] Allard WJ Terstappen LW. CCR 20th Anniversary Commentary: paving the way for circulating tumor cells. Clin Cancer Res (2015) 21:2883–5. 10.1158/1078-0432.CCR-14-2559 26133774

[37] Maltoni R Gallerani G Fici P Rocca A Fabbri F. CTCs in early breast cancer : a path worth taking. Cancer Lett (2016) 376:205–10. 10.1016/j.canlet.2016.03.051 27060205

